# Intraspecific Variation Along an Elevational Gradient Alters Seed Scarification Responses in the Polymorphic Tree Species *Acacia koa*

**DOI:** 10.3389/fpls.2021.716678

**Published:** 2021-11-04

**Authors:** Anna Sugiyama, James B. Friday, Christian P. Giardina, Douglass F. Jacobs

**Affiliations:** ^1^Department of Forestry and Natural Resources, Hardwood Tree Improvement and Regeneration Center, Purdue University, West Lafayette, IN, United States; ^2^School of Life Sciences, Harold L. Lyon Arboretum, University of Hawai'i at Mānoa, Honolulu, HI, United States; ^3^College of Tropical Agriculture and Human Resources, University of Hawai'i at Mānoa, Hilo, HI, United States; ^4^Institute of Pacific Islands Forestry, United States Department of Agriculture Forest Service, Hilo, HI, United States

**Keywords:** elevational gradient, forest restoration, germination, Hawai'i Island, hot water treatment, imbibition, mother tree, scarification index (*SI*)

## Abstract

Physical dormancy in seeds can challenge restoration efforts where scarification conditions for optimal germination and seedling vigor are unknown. For species that occur along wide environmental gradients, optimal scarification conditions may also differ by seed source. We examined intraspecific variation in optimal scarification conditions for germination and seedling performance in koa (*Acacia koa*), which occurs across a wide range of environmental conditions. To evaluate scarification responses, we recorded imbibition percentage, germination percentage, germination time, seedling abnormalities, early mortality, seedling growth, and seedling survivorship. From these, we developed a scarification index (*SI*) that integrates these measures simultaneously. We hypothesized that seeds from lower elevation sources exposed to higher temperatures would have harder seed coats and would require more intense scarification treatments. To test this hypothesis, we repeatedly exposed seeds to hot water differing in temperature and time until seeds imbibed. Supporting the hypothesis, seeds from lower elevation sources generally required more intense scarification, although we found substantial variation among sources. Koa seeds germinated in about a week following imbibition. Boiling seeds (i.e., maintaining at 100°C) was effective for imbibing seeds but it also substantially reduced germination percentages. Repeated exposure to 90 to 100°C water did not reduce germination percentage but decreased seedling performance and increased early mortality. No seeds remained unimbibed after six attempts of boiling germinated whereas seeds remaining unimbibed after 15 attempts of exposure to 90 to 100°C water showed high germination percentages. Abnormalities in seedling development were rare but increased with treatment intensity. Exposure to 100°C water for 1 min overall generated the best *SI* values but the best treatment differed by elevation, and the treatment with the best *SI* was rarely predicted from the highest germination percentages. Seeds that imbibed without any treatment germinated at the same level as manually filed seeds but produced poor seedling quality. Variation in mother tree environments along an elevational gradient can lead to differences in seed coat characteristics, which may explain differing responses to treatments. Scarification treatments affected processes beyond imbibition and germination and using an index like *SI* may improve efficiency by identifying optimal scarification treatments while reducing seed waste.

## Introduction

Forest restoration is often expensive and labor-intensive. Small changes in practice especially during early stages can improve resource use and restoration outcomes while reducing costs. For example, selecting good quality mother trees (source trees) can lead to higher propagation success in nurseries, which in turn can accelerate returns on restoration investment (Hufford and Mazer, 2003; Burrows et al., 2009; Jalonen et al., 2018). Yet, fewer than half of global forest and landscape restoration projects record their seed sources (Jalonen et al., 2018), with use of poor-quality seeds often leading to waste of resources. Seed sourcing and availability remains a major challenge for many restoration projects (Wuethrich, 2007; Broadhurst et al., 2008; Merritt and Dixon, 2011), while demand for seeds is increasing (Burrows et al., 2009; Merritt and Dixon, 2011; Koskela et al., 2014). Thus, a lack of attention to seed source can lead to higher restoration costs, seed and greenhouse waste, and compromise future restoration success.

For species that require scarification to break physical dormancy, identifying optimal treatments can improve resource use and long-term success. For example, the Fabaceae (legumes), one of the most common families in agriculture and in natural ecosystems (Graham and Vance, 2003), contains many species with hard seeds, which poses distinct challenges to those handling seeds (Lebedeff, 1947). Various methods have been tested to identify effective scarification treatments for a wide variety of legume species, including the pantropical genus *Acacia*, with optimal methods varying substantially among species (Doran et al., 1983; Ghassali et al., 2012). Less known is the extent to which intraspecific variation requires genotype specific scarification conditions, as optimal scarification requirements may vary among seeds sources. Despite the increasing awareness that intraspecific variations are important in ecological studies (Hufford and Mazer, 2003; Siefert et al., 2015), intraspecific variation in physical dormancy has been studied primarily in agricultural non-woody species (Hoyle et al., 2011; Hudson et al., 2015), with few studies examining intraspecific variation in scarification optima for forest species.

Seed hardness can be one driver of variation in scarification requirements, with hardness differing by seed lot, plant genotype, seed morphology, population, year, climate, storage time, or a mix of these factors even for a given species (Pathak et al., 1980; Smith, 1988; Cervantes et al., 1996; Boyle and Hladun, 2005). Environments of mother trees also affect physical dormancy of seeds. Higher temperature and lower rainfall or relative humidity, both of which help to reduce moisture content in seeds, tend to promote seeds with stronger physical dormancy, by increasing seed coat thickness and reducing water permeability, even for the same species and site (Hudson et al., 2015; Liyanage and Ooi, 2015; Jaganathan, 2016).

Effective scarification treatments are generally targeted as a condition that allows rapid and synchronous germination of seeds, but identifying optima requires good understanding of what impacts scarification treatments have on different processes, from imbibition to seedling performance. For example, a treatment that maximizes germination may not maximize the efficiency or the number of vigorous seedlings. Hot water is a relatively easy, safe, and low-cost scarification method (Doran et al., 1983) that is commonly used for hard-seeded species like *Acacia*. Exposure to near-boiling hot water for a short time may safely treat the seeds with little detrimental effect but may only imbibe a small proportion of seeds. If many seeds require repeated exposure, efficiency declines significantly. On the other hand, exposure to boiling water may result in high imbibition percentage but impair seed or seedling survival and sound development (Clemens et al., 1977; Burrows et al., 2009). However, the effects of scarification treatments on different processes and the consequence of repeated exposure of unimbibed seeds to hot water are largely unknown.

Here, we tested whether optimal scarification conditions differ for seeds from mother trees growing across a steep elevational gradient in an *Acacia* species on Hawai'i Island. We specifically tested whether elevations of mother trees would predict intraspecific variation in the effectiveness of scarification. Using differing hot water treatments repeatedly until seeds imbibed, we recorded: (1) imbibition percentage, (2) germination percentage, (3) germination time (median number of days to germination), (4) seedling abnormalities, (5) early mortality, (6) seedling growth, and (7) seedling survivorship. We used these measurements and developed a scarification index (*SI*) that integrates these processes simultaneously to test the hypothesis that seeds from lower elevations with higher temperatures would require more intense scarification treatments (i.e., higher temperature, longer exposure time, more repeated scarification treatments) to break dormancy.

## Materials and Methods

### Study Species

To test whether the optimal scarification condition differed by elevation of mother trees, we used *Acacia koa* (Fabaceae; hereafter koa), which is a largely outcrossing (Daehler et al., 1999), evergreen pioneer tree species endemic to major islands of Hawai'i. Koa seeds have a hard seed coat that is impermeable to water and imposes physical (exogenous) dormancy (Baskin and Baskin, 2014), and require scarification of the seed coat to trigger germination (Allen, 2002; Wilkinson and Elevitch, 2003; Elevitch et al., 2006). Extensive areas of koa forests, especially in more mesic higher elevation areas, have been converted to alternative land uses via logging, grazing, and, more recently, fire and introduced pests and diseases, such as koa wilt disease (*Fusarium* spp.). These threats have severely reduced populations at lower elevations (Gardner, 1980; Wilkinson and Elevitch, 2003; Elevitch et al., 2006; Dudley et al., 2020). In the past decade, the native koa moth (*Scotorythra paludicola*) also caused wide spread defoliation of koa on Hawai‘i Island and significant koa mortality (Stein and Scowcroft, 1984; Banko et al., 2014). With its high timber value and high ecological and cultural importance, there has been a growing interest in restoring koa to its previous range (Whitesell, 1984; Scowcroft et al., 2004; Pejchar et al., 2005). Koa can grow across wide elevation (24-2,440 m) and precipitation (635-5,080 mm) ranges (Whitesell, 1984; Wilkinson and Elevitch, 2003). Owing to such broad range of environmental gradients, koa seeds express wide variation in morphology (Whitesell, 1984; Daehler et al., 1999; Ishihara et al., 2017), which may translate into differences in optimal scarification conditions.

### Sources of Seeds

Between September 12 and 17, 2018, we collected seeds from 17 trees located between 333 m and 2,038 m a.s.l. in State Forest Reserves along the east side of Saddle Road, which runs across Hawai'i Island, and on private land north of Saddle Road. We collected fruits from branches using an extension pole pruner, and from the ground when there were no other fruiting conspecific trees nearby. Because of the overall low fruit crop in 2018 (due apparently to a wet spring and summer), we also relied on banked seeds at the University of Hawai'i at Mānoa College of Tropical Agriculture and Human Resources Komohana Research and Extension Center in Hilo. We selected seeds from an additional 22 trees growing between 308 m and 2,103 m a.s.l. (collected between 2014 and 2016 on Hawai'i Island), including one tree that we were able to resample in 2018 (Supplementary Table 1). Koa seeds are orthodox and can remain viable for 25 years under optimal conditions (Judd, 1920). Because storing seeds for 2–4 years may result in differences not related to elevation or treatment effects, we tested whether stored vs. newly collected seeds exhibited differences in imbibition percentage, germination percentage, germination time, seedling abnormalities, early mortality, seedling growth, or seedling survivorship. We detected no storage effects on any of the measures (data not shown), so we analyzed all seeds together. After extracting seeds, we kept visibly intact seeds in labeled, airtight resealable plastic bags. Seed bank seeds were vacuum sealed for long-term storage and were kept in a refrigerator at 8°C. We kept all the seeds in a refrigerator until shipping them from Hawai'i to Indiana (Purdue University) where we conducted all the experiments. Once seeds arrived in Indiana, we kept seeds in a refrigerator at 4°C for up to 1 month at which point they were sown.

### Scarification Experiments

We conducted two sets of experiments: (1) a pre-trial of potential scarification treatments using seeds collected from a single tree (Supplementary Table 1), and (2) a full experiment using a range of scarification methods and treatment times. For the latter, we used all 38 mother trees along a wide elevational gradient to test for intraspecific variation in response to one of the six treatments using hot water. We also used three mother trees each from three elevational ranges to test intraspecific variation in optimal scarification condition by exposing these seeds to all treatments. For both the pre-trials and the full experiments, we treated seeds with hot water, which allowed us to scarify large quantities of seeds without special equipment or chemicals. First, we discarded seeds with visible damage that were present after collection (e.g., mold, insect holes), seeds that had already imbibed, and seeds that floated on the water. We used latex gloves when handling seeds to prevent the potential spread of fungal spores that cause koa wilt, and sterilized seed surface using 0.6% sodium hypochlorite solution for 2 min.

### Pre-Trials

To identify the range of temperatures and exposure times that would potentially maximize imbibition while minimizing seed damage, we conducted scarification pre-trials in October 2018. For these pre-trials, we used purchased seeds collected from a single tree (1,372 m a.s.l.) in Kailua Kona (Supplementary Table 1). Based on previously recommended conditions (Wilkinson and Elevitch, 2003; Elevitch et al., 2006) and our experience, we exposed seeds at three different temperatures (90, 95, and 100°C) each for three different times (1, 2, and 3 min). We also manually scarified the seeds using a metal file on the lateral side of the seed as a ‘control' not treated by hot water (*N* = 20 seeds each treatment). We poured deionized water heated to the specified temperature (except for control) to seeds at a volume ratio of about 10 parts water to one-part seed, while consistently stirring the seeds. After the specified time, we immediately rinsed the seeds with room temperature deionized water (~21°C) and soaked the seeds overnight. The following day, we recorded the number of imbibed seeds, which was visually conspicuous (Supplementary Figure 1). We manually filed the remaining unimbibed seeds, and soaked them in room temperature water overnight, which imbibed all seeds. The following day, we sowed them in the greenhouse. Due to overall low imbibition percentages (20–40%) after one attempt of exposure to hot water, we followed up by adding more intense treatments. Using the remaining seeds, we boiled seeds for 1 min (i.e., maintained at 100°C as opposed to pouring over 100°C water). We also exposed seeds at 90°C for 1 min but this time, repeated this procedure until the seed imbibed instead of manually filing the seed after one attempt (*N* = 10 seeds each treatment). For both of these follow-up trials, we exposed seeds at a volume ratio of about 50 parts water to one-part seed.

### Full Experiments

We conducted full scarification experiments using a total of 4,619 seeds (Supplementary Table 1) between late November and December 2018. To test for intraspecific variation in response to the same scarification treatment among mother trees, we boiled seeds from all 38 trees for 0.5 min (*N* = ~40 seeds for most trees except for six trees used for another study which had 240 additional seeds per tree; Supplementary Table 1), separately for one tree collected in two different years, based on the imbibition and germination results from the pre-trials.

To test for optimal scarification conditions for mother trees differing in elevation, we randomly selected three mother trees with sufficient sample size each for low (300–350 m), mid (700–950 m), and high (2,000–2,150 m) elevations (Supplementary Table 1). Elevation ranges were not continuous because koa trees are largely absent between 1,000 and 2,000 m on the east side of the Hawai'i Island (Baker et al., 2009). We exposed seeds from the nine selected trees to water at one of three temperatures (90, 95, and 100°C) for 1 min, or boiled seeds for one of two additional exposure times (1 and 2 min, in addition to 0.5 min) at a volume ratio of about 20 parts water to one-part seed (*N* = ~30 seeds each). In addition to these six treatments using hot water, we soaked intact seeds in room temperature water overnight and recorded seeds that imbibed without any treatment (“not treated”). Then, we manually filed the remaining soaked but unimbibed seeds, which were our ‘control,' untreated by hot water. After each treatment, we let the treated seeds soak in room temperature water overnight, and recorded imbibition the following day. When seeds were partially imbibed (Supplementary Figure 1), we recorded them as imbibed and did not apply further scarification treatment to avoid potential damage. When seeds did not imbibe, we repeated each treatment up to 15 times. After the 15th attempt, we manually filed any remaining seeds that did not imbibe (hot water treatment + manual filing). As for other treatments, we sowed them after soaking them in room temperature water overnight, which imbibed all seeds. In any experiment, we did not sow seeds that were visually unimbibed. For all experiments, we randomly allocated seeds from each tree across all treatments to equally distribute any variation in seeds.

### Germination Experiments

Once seeds were at least partially imbibed, we sowed them the same day imbibition was recorded. We placed them on flat trays with BM2 Seed Germination and Propagation Mix (mix of peat moss, perlite, vermiculite, dolomitic and calcitic limestone, wetting agent, and fertilizer starter charge; Berger, Saint-Modeste, Quebec) in the Horticulture and Landscape Architecture greenhouse at Purdue University, and labeled each batch (source tree, treatment, the number of attempts) separately. The greenhouse was set to maintain 25.6°C during the day, 22.2°C at night, and relative humidity below 80%. To promote plant growth during short-day times, photoperiod was set to 14 h where natural light was supplemented using 600 W high pressure sodium lamps (photosynthetic photon flux of ~100 umol m^−2^ s^−1^ photosynthetic active radiation) until March 2019. After which, they were then supplemented less depending on the weather until May 2019, when no artificial lights were used thereafter. We randomly moved the location of trays to account for spatial heterogeneity within the greenhouse, manually sub-irrigated seedbeds, and recorded germination (defined as extension of hypocotyl and exposure of green cotyledons) every day for 6 weeks to obtain germination rates. After three months, we also recorded the total number of germinants to capture any delayed germination.

### Seedling Assessments

Following germination, we transplanted seedlings (mean 40 ± 25 days after sowing seeds) into D40L Deepots (Stuewe & Sons, Inc., Tangent, Oregon) filled with the Sungro Professional Growing Mix Fafard® 52 Mix Metro-Mix® 852 (mix of bark, peat moss, perlite, dolomite lime, wetting agent, and silicon; Sun Gro Horticulture, Agawam, Massachusetts). We labeled each seedling with a unique identifying number to which batch information was tagged and randomly placed them into slots within a randomly selected rack, and randomly placed them within the greenhouse, and recorded the rack number to account for spatial heterogeneity within the greenhouse. Once we transplanted seedlings, we manually watered seedlings daily and fertilized them weekly with 150 ppm N Peters Professional® 20-3-19 Petunia Special with black iron fertilizer (7.8% NH_4_+, 12.2% NO_3_-, 3% P_2_O_5_, 19% K_2_O, 1.6% Mg, 2.8% S, 0.0026% B, 0.0024% Cu, 0.2% Fe, 0.05% Mn, 0.011% Mo, 0.052% Zn; ICL Specialty Fertilizers, Dublin, Ohio). To assess the potentially negative effects of hot water treatments on seedling performance, we also recorded abnormalities (e.g., undeveloped fine roots, uprooting, limited leaf expansion beyond cotyledons, albinos) at the time of transplant. When seedlings died within three months before being transplanted, we recorded the event as early mortality. Finally, we recorded basal diameter, height, number of phyllodes, which are flattened non-true leaves that serve as leaves in older koa plants (Rose et al., 2019), and survivorship of all transplanted seedlings at the end of this study, which varied by seedling from end of July to August 2019. We set a fixed window of at least 19 weeks after being transplanted, and calculated seedling growth as (size_n+1_ - size_*n*_)/(record date - sowed date).

### Scarification Index (*SI*)

For restoration, an optimal scarification treatment can be defined as a condition that maximizes imbibition percentage, germination percentage, seedling growth, and survivorship, while minimizing days until germination, abnormalities, and early mortality. To consider them simultaneously, we developed a scarification index (*SI*) that combines these measures as follows: *SI* = *i* × *g* × *d* × *a* × *m* × *h* × *s* where *i* is proportion of seeds imbibed, *g* is proportion of seeds germinated, *d* is normalized median days until germination [1 – (median day - minimum median day)/(maximum median day - minimum median day)], *a* is 1 - abnormality (i.e., proportion of normal seedlings), *m* is 1 - early mortality, *h* is normalized seedling size [(size - minimum size)/(maximum size - minimum size)], and *s* is seedling survivorship. *SI* and variables *i, g, d, a, m, h*, and *s* all range between 0 and 1, and optimal scarification treatment will result in the highest *SI* value. For each process, we calculated coefficient of variation (CV) across treatments to determine the impact of scarification treatments on each process. A large CV suggests that the type of scarification treatment has a large, differentiating impact on that process.

### Statistical Analyses

We tested imbibition percentage, germination percentage, germination time, abnormalities, early mortality percentage, seedling growth, and seedling survivorship separately as response variables. We used linear mixed models (binary distribution and logit link for imbibition, germination, abnormalities, early mortality, and seedling survivorship, and Gaussian distribution and identity link for seedling growth) where treatment and elevation (or categorical elevation range), and their interactions were fixed effects, and mother tree and rack location (for seedling growth and survivorship only) were random effects. When interaction terms were not significant, they were excluded from subsequent model runs. For imbibition and germination percentages only, we used linear mixed models with treatment as a fixed effect and conducted pair-wise comparisons of estimated marginal means between treatments per elevation range. We also used linear mixed models when we tested the effects of partial and full imbibition because it was only recorded at the batch level (i.e., the number of attempts per treatment per tree) and not at the individual seed level. For analyses involving processes before seedlings emerge (i.e., imbibition, germination, and germination time), we excluded the additional 240 seeds from our analyses because these seeds were manually filed when they did not imbibe after the first attempt, and the denominator differed between the first and the first through the 15th attempts combined.

To study the effects of the number of repeated exposures to hot water, we categorized the number of attempts into (1) the first attempt and (2) multiple attempts (second through 15th attempt without manual filing combined) to balance the sample sizes between the categories. We also ran the same models for data after only one attempt and the first through 15th attempts combined (without seeds that were subjected to manual filing when they did not imbibe after the 15th attempts). When we used non-categorical elevation and the number of attempts, we standardized them before the analyses. We either dropped a fixed effect or used categorical variables instead when multicollinearity existed among fixed effects (generalized variance inflation factor^1/(2df)^ > 2) and selected the best model using Akaike information criterion. When we were interested in linear relationships between variables, we conducted linear regressions. We used all trees whose seeds were boiled for 0.5 min when we were interested in testing for intraspecific variation across trees from an environmental gradient in effects other than treatment.

As for days until germination, although a large proportion (98.1%) of seeds germinated within six weeks, during which we recorded germination daily, we did not always have the exact germination dates for seeds that germinated after the said six weeks. Thus, we used median days until germination for each batch. We also analyzed these results using days until first germination but there was no difference in overall pattern and the median value for the number of days until germination had the greatest sample size. Thus, we only report results using median days until germination for germination time. We conducted median tests among categorical variables because data were over-dispersed. We performed all statistical analyses using R 3.6.2 (R Core Team, 2019) with packages ‘lme4' ver. 1.1.26 (Bates et al., 2015) and ‘lmerTest' ver. 3.1.3 (Kuznetsova et al., 2017) for linear mixed models, ‘emmeans' ver. 1.5.4 (Lenth, 2021) for pair-wise comparisons among treatments, and ‘agricolae' ver. 1.3.3 (de Mendiburu, 2020) for median tests. When normality assumption was not met in linear mixed models, we used arcsine transformation. We only report results from up to after 15 attempts combined, standard deviation as errors, and adjusted *R*^2^ as *R*^2^, unless otherwise mentioned.

## Results

### Imbibition Percentage

**Figure 1:**
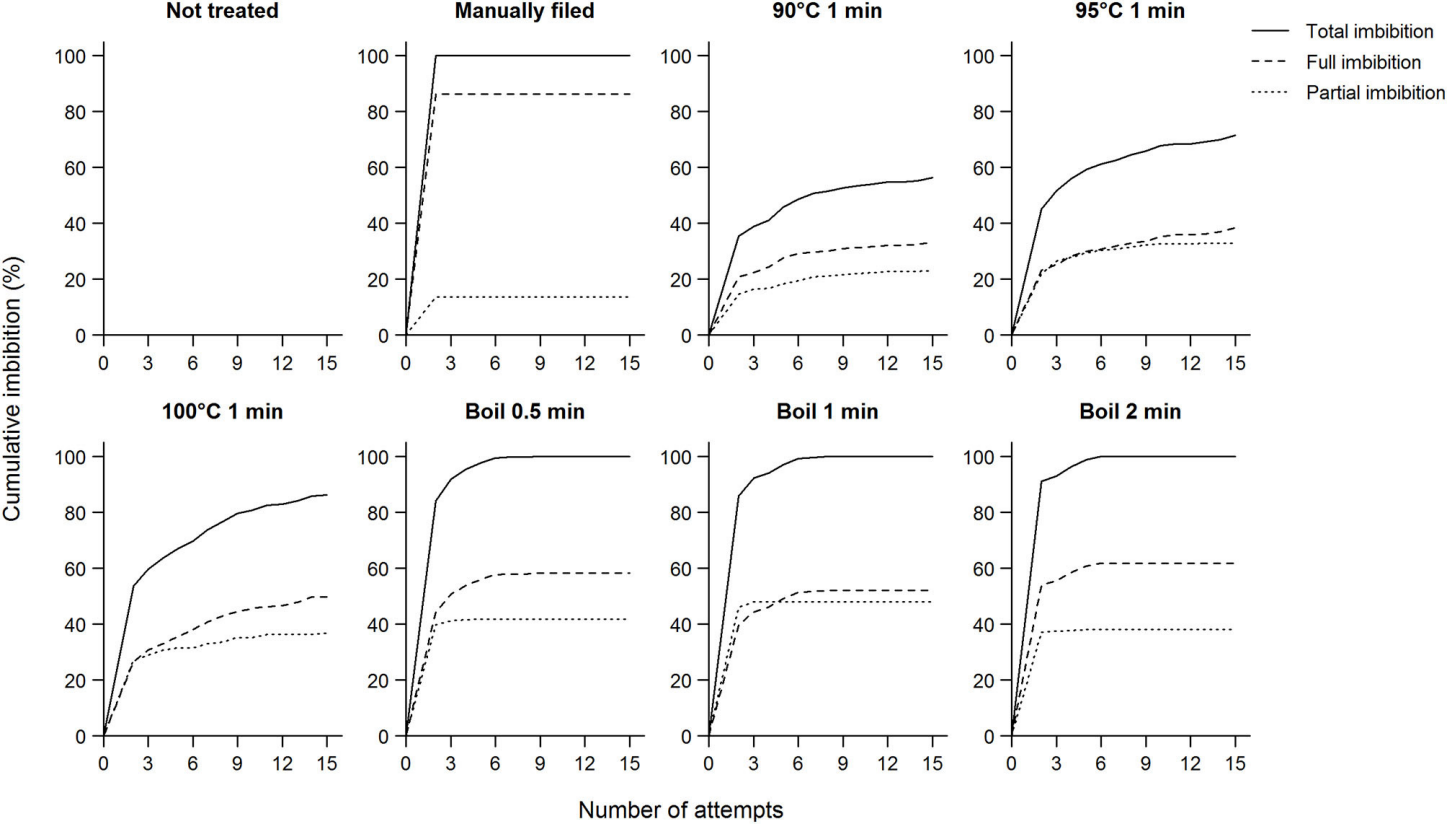
Cumulative imbibition percentage of koa seeds and the number of attempts each treatment took for seeds to imbibe shown separately for whether seeds were fully imbibed or not. Total imbibition is the sum of full and partial imbibition (Supplementary Figure 1). As for untreated seeds, we soaked seeds in room temperature water overnight without manual filing; only a small proportion of these seeds imbibed and the results are not visible in the subpanel. When seeds did not imbibe after 15 attempts, we manually filed them, which resulted in 100% imbibition (these results are excluded here). Data for each subpanel are for seeds from nine trees subjected to all scarification treatments.

Koa seeds did not readily imbibe without treatment, and some seeds did not imbibe even after 15 applications of a treatment (Figure 1). Without treatment (either manual filing, exposure to hot water, or boiling), only 3.0% of all seeds imbibed after soaking in room-temperature water overnight (Table 1). When exposed to 90 to 100°C water for 1 min, from 21.3 to 33.3% of seeds imbibed after the first attempt, which increased from 56.3 to 86.3% after up to 15 attempts. Increasing the treatment temperature (exposure time for boiling treatments) and the number of attempts generally increased imbibition percentage.

**Table 1:** Imbibition, germination, germination time, abnormalities, early mortality, seedling height growth, and seedling survivorship across nine trees subjected to all scarification treatments after up to 15 attempts combined.

**Treatment**	**Imbibition (%)**	**Germination (%)**	**Germination time (days)**	**Abnormalities (%)**	**Early mortality (%)**	**Seedling growth (cm/day)**	**Seedling survival (%)**
Not treated	3.0	62.5	9.5	0.0	20.0	0.21 ± 0.07	100.0
Manually filed	100.0	56.1	6.0	0.0	11.6	0.37 ± 0.19	92.3
90°C 1 min	56.3	78.8	6.0	0.0	14.2	0.38 ± 0.21	89.9
95°C 1 min	71.4	73.3	7.0	0.5	8.0	0.38 ± 0.18	92.1
100°C 1 min	86.3	68.2	6.0	1.1	10.3	0.41 ± 0.19	91.6
Boil 0.5 min	100.0	32.3	7.0	2.1	12.4	0.45 ± 0.20	77.9
Boil 1 min	100.0	26.0	6.5	4.3	8.6	0.48 ± 0.18	84.1
Boil 2 min	100.0	18.9	6.0	27.5	3.9	0.49 ± 0.20	88.0

**Figure 2:**
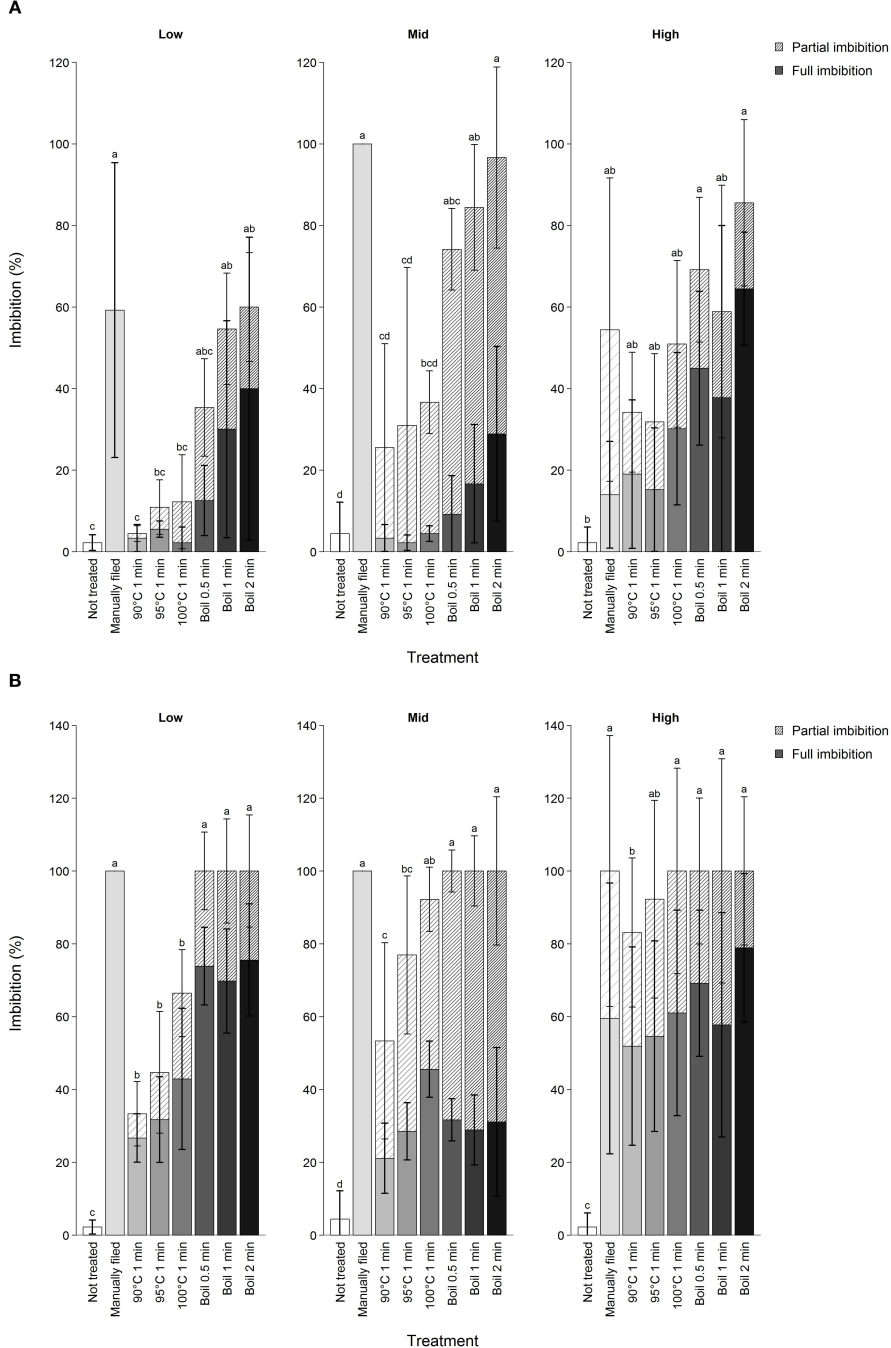
Imbibition percentage of koa seeds by different scarification treatment after **(A)** first treatment attempt and **(B)** first through up to 15 attempts combined excluding those that were subjected to manual filing afterwards. Scarification treatments are ordered from the least intense to the most intense from left to right, indicated by the darkness of the bar color. Hatched and filled bars show partial and full imbibition, respectively. Error bars are standard deviation shown separately for full (bold) and partial imbibition for seeds from three trees per elevation range. Different letters indicate difference in total imbibition percentage among treatments for each elevation range at α = 0.05.

Treatment effectiveness varied by elevation of the mother tree. Overall, seeds from the lower elevation range required more intense scarification for a given treatment, supporting our hypothesis (Figure 2). Thus, the likelihood of seeds not imbibing after the 15th attempt was higher in seeds from low elevation than other elevations (estimate = −1.5 ± 0.24, *Z* = −6.4, *P* < 0.01). Despite such trends, imbibition was not predictable simply from elevation of mother trees because of significant elevation by treatment interactions (data not shown). However, this was true only in 90 to 100°C water treatments and when all attempts were considered (Supplementary Figure 2). No elevational differences in imbibition were found prior to applying treatment or from manual filing.

Substantial inter-tree variation existed for imbibition likelihood. Comparing seeds from all trees subjected to boil 0.5 min, imbibition percentage after the first attempt varied from 20.0 to 100%. Whether seeds likely imbibe fully or partially was also determined by the identity of mother tree (full imbibition: 0–82.9%, partial imbibition: 0–85.0%) but partial imbibition was more common after the first attempt than after multiple attempts (estimate = 36.8 ± 3.5, df = 137.8, *t* = 10.7, *P* < 0.01).

### Germination Percentage

Increasing the intensity of scarification treatment increased imbibition percentage, but it also reduced germination percentage (Table 1). Although seeds that imbibed before any treatment could have been seeds that were compromised, they were not necessarily of poor quality in terms of germination and did not differ from manually filed seeds.

**Figure 3:**
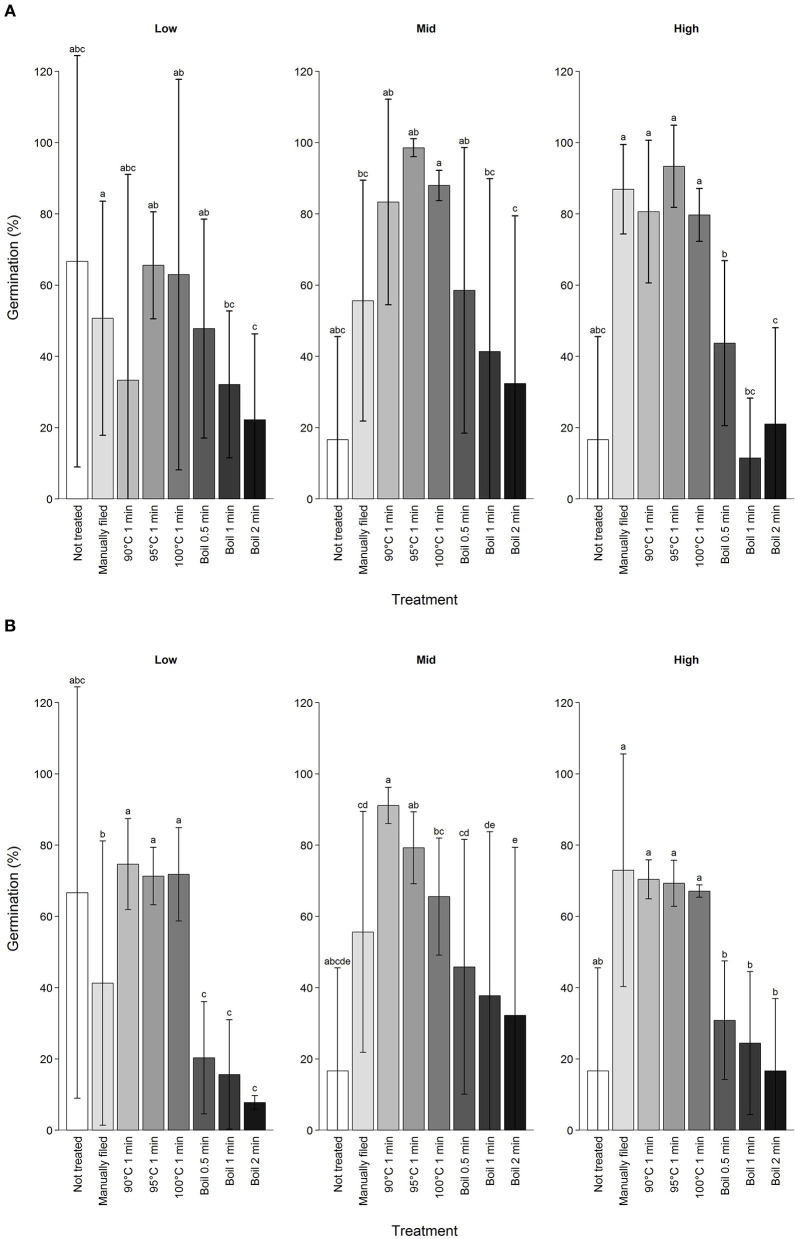
Germination percentage of koa seeds by different scarification treatment after **(A)** first treatment attempt and **(B)** first through up to 15 attempts combined excluding those that were subjected to manual filing afterwards. Scarification treatments are ordered from the least intense to the most intense from left to right, indicated by the darkness of the bar color. Error bars are standard deviation for seeds from three trees per elevation range. Different letters indicate difference in total germination percentage among treatments for each elevation range at α = 0.05.

Manual filing did not result in the highest germination percentage among different scarification treatments (Figure 3). Overall, any of the seeds subjected to 90 to 100°C water treatments resulted in higher germination percentages than the manual filing treatment (estimate = 0.80 ± 0.16, *Z* = 5.1, *P* < 0.01) but effect size decreased with elevation (estimate = −0.76 ± 0.16, *Z* = −4.8, *P* < 0.01). On the other hand, boiling seeds substantially reduced germination percentage compared to the manual filing treatment (estimate = −1.4 ± 0.16, *Z* = −8.7, *P* < 0.01) with effect size increasing with elevation (estimate = −0.49 ± 0.15, *Z* = −3.2, *P* < 0.01). Despite such trends, scarification treatments had stronger effects on germination percentage than elevation, and germination was not directly predictable from elevation of mother trees except for manually filed seeds (Supplementary Figure 3). Comparing seeds from all trees subjected to boiling for 0.5 min, germination percentage after the first attempt varied from 23.1 to 100% and 15.0 to 90.0% after up to 15 attempts.

**Figure 4:**
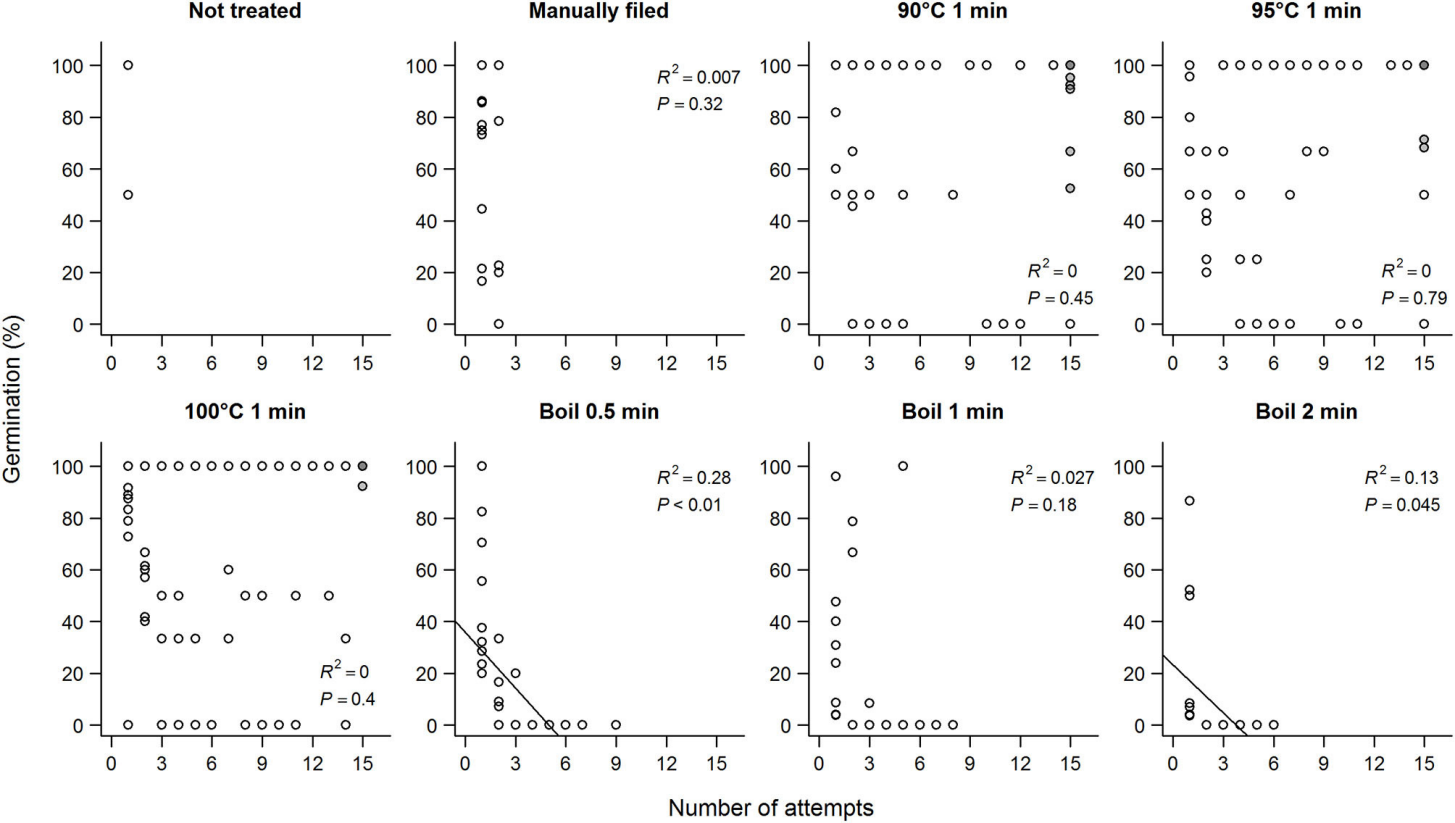
Germination percentage of koa seeds and the number of attempts each scarification treatment took for seeds to imbibe. Each circle represents a mother tree, and the filled circles are seeds that did not imbibe after the 15th attempt and were subjected to manual filing afterwards. Data for each subpanel are for seeds from nine trees subjected to all scarification treatments.

Repeated exposure to hot water for 1 min did not reduce germination percentages unless seeds were boiled (Figure 4). In fact, seeds that did not imbibe after 15 attempts and were subjected to manual filing showed higher germination percentage compared to seeds that imbibed with fewer than 15 attempts and no filing (87.7 vs. 67.6%; estimate = 1.6 ± 0.25, *Z* = 6.3, *P* < 0.01) for the same treatments (90–100°C 1 min). In contrast, no seeds germinated after the sixth boiling for 0.5 min, or the fourth boiling for 1 or 2 min. Overall, seeds that imbibed after the first attempt germinated more than seeds that imbibed after multiple attempts (60.7 vs. 36.3%) although being imbibed after the first attempt on its own did not predict germination. Because seeds were more likely to imbibe only partially after the first attempt and fewer seeds germinated when imbibed after multiple attempts, seeds that were fully imbibed overall showed lower germination percentage (estimate = −0.0050 ± 0.00097, df = 313.0, *t* = −5.1, *P* < 0.01).

### Germination Time

**Figure 5:**
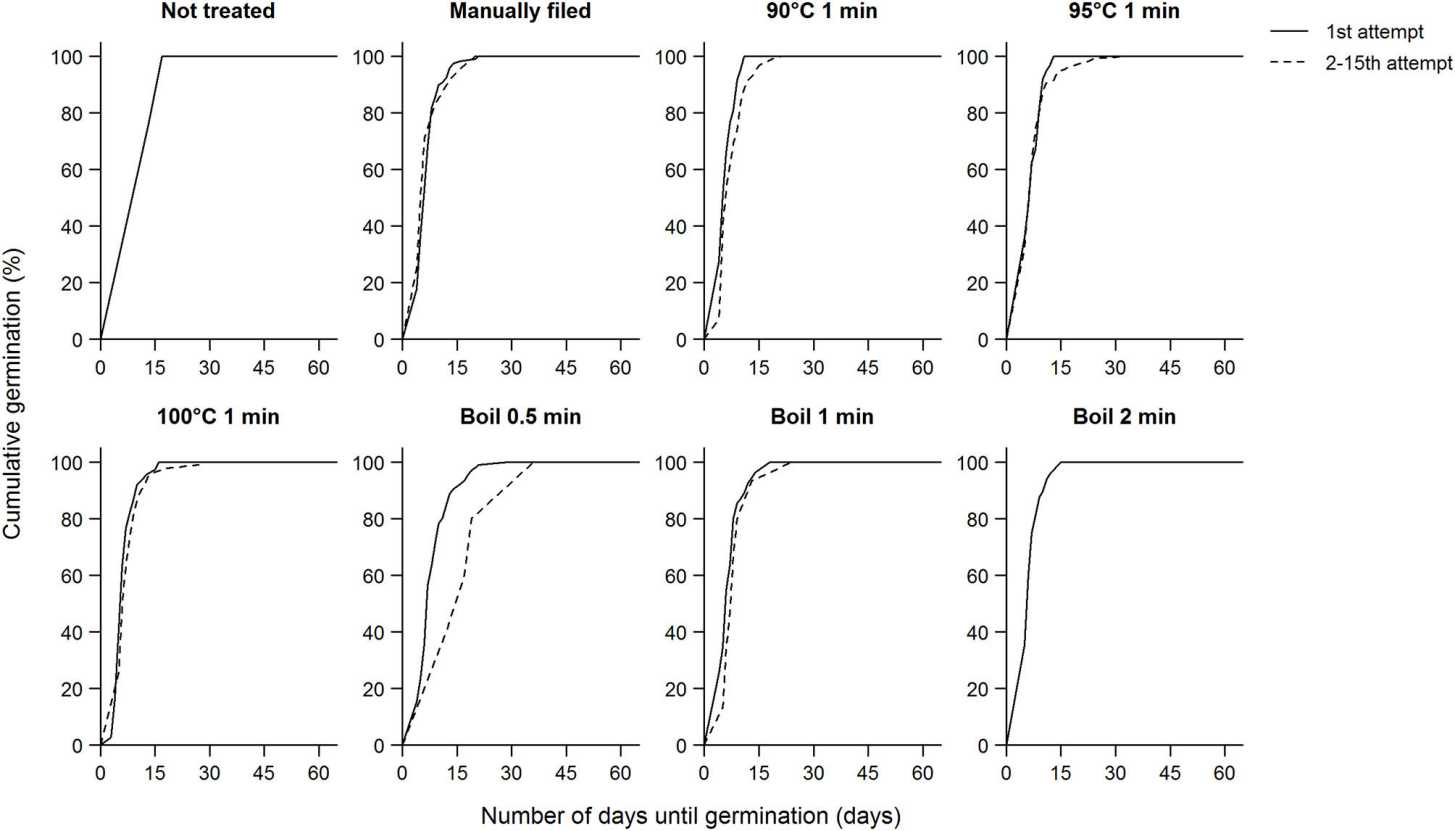
Cumulative germination percentage of viable koa seeds after subjected to each scarification treatment and the number of days until germination shown separately for the first attempt and up to the second through the 15th attempts combined. There was no difference between the first attempt and the second through the 15th attempt combined except for boil 0.5 min (χ^2^ = 6.2, df = 1, *P* = 0.01). Data for each subpanel are for seeds from nine trees subjected to all scarification treatments.

In addition to expediting germination, scarification also allows uniform germination, and most koa seeds germinated within a fairly narrow window irrespective of treatment (Table 1). On average, koa seeds germinated in 7.3 ± 3.9 days (median: 6 days) across all trees, ranging from a range of 5.1 ± 1.2 days to 11.3 ± 12.1 days (median: 5–8 days). Overall, there was no difference in germination time between seeds that germinated after the first attempt and after multiple attempts except in the boil 0.5 min treatment in which seeds that imbibed after multiple attempts germinated slower than those imbibed after the first attempt (Figure 5). This pattern was driven by low elevation seeds that imbibed after multiple attempts, which led to significant elevation effects on germination time (χ^2^ = 12.4, df = 2, *P* < 0.01). Similarly, because of such delay in germination time when boiled for 0.5 min multiple times, treatment effects on germination time were significant (χ^2^ = 18.8, df = 7, *P* < 0.01), driven by the difference between the boil 0.5 min treatment and manual filing, 100°C 1 min, and boil 2 min treatments. Although seeds that were manually filed after 15 attempts showed higher germination percentages, these seeds did not germinate faster. Similarly, whether seeds were partially or fully imbibed did not appear to affect germination time.

### Abnormalities

We found abnormalities in only 1.2% of all koa germinants (*N* = 2,104). Of seedlings that showed abnormalities, 96.2% were from seeds that imbibed after the first attempt. Although sample size was too small to run most statistical tests, the likelihood of a seedling being abnormal generally increased with the intensity of treatment (Table 1). No seeds exposed to 90°C for 1 min or seeds not treated with hot water showed abnormalities. Abnormalities were observed in seedlings from only five mother trees (0–8.2% across trees). These trees were mostly concentrated in mid elevations, with none at lower elevations. However, abnormalities were not related to elevation or the number of attempts.

### Early Mortality

Of all germinated koa seedlings, we found that 9.2% died within the first three months, and 57.0% of all early mortality was associated with seedlings that germinated after the first attempt. Early mortality percentage was highest for seedlings from untreated seeds across treatments (Table 1), but treatment overall had no effect on early mortality. Although treating seeds multiple times did not increase early mortality percentage in seeds subjected to all treatments, repeated attempts increased the likelihood of early mortality across all seedlings from the boil 0.5 min treatment (estimate = 0.48 ± 0.08, *Z* = 5.9, *P* < 0.01).

Seedlings from high elevation trees showed significantly lower early mortality than seedlings from low or mid elevation trees (estimate = −1.3 ± 0.41, *Z* = −3.0, *P* = 0.02). However, seedlings that showed early mortality varied substantially, from 0 to 71.4% and early mortality was not linearly related to elevation.

### Seedling Growth

On average, koa seedlings were 92.3 ± 46.3 cm in height and 7.4 ± 3.1 mm in basal diameter after an average 7.0 ± 0.8 month growing period. Seedling growth varied substantially by mother tree, ranging from 0.22 ± 0.13 to 0.66 ± 0.11 cm day^−1^ in height and from 0.19 ± 0.01 to 0.47 ± 0.01 mm day^−1^ in basal diameter across all trees. Whether seedlings had phyllodes or not also varied substantially across all trees used in full experiments, from 0 to 4.9 ± 9.2 phyllodes per seedling. We only report results using seedling height growth for the entire study period because seedlings from some trees had no phyllodes, and because seedling height and basal diameter (*R*^2^ = 0.78, *P* < 0.01), their growth (*R*^2^ = 0.74, *P* < 0.01) were highly correlated.

In contrast to treatment effects on germination percentage, increasing the intensity of the treatments led to overall increased seedling growth (Table 1). Although all treatments, including boiling seeds, had positive effects on seedling height (data not shown), height growth decreased with increasing number of attempts (estimate = −0.021 ± 0.010, df = 639.5, *t* = −2.1, *P* = 0.04), and elevation had no effect when all three factors were considered. However, when comparing all trees subjected to boil 0.5 min treatment, both elevation (estimate = −0.051 ± 0.019, df = 30.8, *t* = −2.7, *P* = 0.01) and the number of attempts (estimate = −0.039 ± 0.010, df = 590.0, *t* = −3.9, *P* < 0.01) had negative effects on seedling growth.

Seeds that did not imbibe after 15 attempts and were subjected to manual filing resulted in seedlings that had lower growth compared to those from seeds that had imbibed within 15 attempts (0.37 ± 0.20 vs. 0.40 ± 0.19 cm day^−1^; estimate = −0.078 ± 0.026, df = 396.9, *t* = −3.1, *P* < 0.01) although their germination percentages were higher. This trend increased with elevation, as evidenced by the significant interaction between elevation and whether seeds imbibed after 15 attempts or not (estimate = −0.071 ± 0.030, df = 398.9, *t* = −2.4, *P* = 0.02). However, elevation alone had no effect. Although we calculated seedling growth based on the date when seeds were sown, and not on the date when they germinated, we found no effect of germination time on seeding growth.

### Seedling Survivorship

We found that 86.1% of all transplanted seedlings (*N* = 1,679) survived to the end of the study, with survivorship ranging from 49.3 to 100% across mother trees, and from 77.9 to 100% across treatments (Table 1). Seedling survivorship was not affected by any treatment, elevation, or the number of attempts. However, for seeds that did not imbibe after 15 attempts and were subjected to manual filing, elevation was negatively related to seedling survivorship (estimate = −0.170 ± 0.006, *Z* = −28.5, *P* < 0.01), and survivorship was lower than seedlings from seeds that imbibed before 15 attempts (31.1% vs. 60.0%; estimate = −0.604 ± 0.006, *Z* = −101.3, *P* < 0.01). This trend increased with elevation (estimate = −1.60 ± 0.006, *Z* = −267.7, *P* < 0.01). Comparing all trees subjected to the boil 0.5 min treatment, survivorship decreased with increasing number of attempts (estimate = −0.688 ± 0.002, *Z* = −392, *P* < 0.01), and this trend again increased with increasing elevation (estimate = −0.248 ± 0.002, *Z* = −133, *P* < 0.01) although elevation alone increased seedling survivorship (estimate = 1.055 ± 0.003, *Z* = 390, *P* < 0.01). Finally, survivorship at the tree level was not related to abnormalities or early mortality.

### Scarification Index (*SI*)

We obtained the following best to worst ranking for scarification treatments based on *SI*: 100°C 1 min > manually filed > 95°C 1 min > boil 0.5 min > 90°C 1 min > boil 1 min > boil 2 min > not treated (Table 2). When restricted to after only one attempt, manually filing seeds ranked the best but the ranking for the rest of the treatments was consistent. We could not calculate normalized median days until germination for untreated seeds because there was no difference between the maximum and minimum median days due to a relatively small sample size. Thus, we also calculated *SI*_m_, which was *SI* using a mean of normalized median days until germination across all treatments for untreated seeds. Both *SI* and *SI*_m_ showed identical rank order across treatments. When we evaluated treatments separately by elevation range of seeds, the rank order of *SI* and *SI*_m_ changed from the rank order of all elevation ranges. Seeds subjected to 100°C 1 min, 0.5 min boiling, and manual filing ranked the best for low, mid, and high elevation, respectively. In contrast, untreated seeds ranked always the least due to low imbibition and seedling growth.

**Table 2:** Scarification index (*SI*), values for each constituent process, and the treatment rank for koa seeds from nine trees subjected to all scarification treatments, and mother trees separated by elevation range (all after up to 15 attempts combined).

**Treatment**	**Imbibition**	**Germination**	**Germination time**	**Abnormalities**	**Early mortality**	**Growth**	**Survival**	***SI***	***SI*_**m**_**	***SI* rank**	***SI*_**m**_ rank**
Not treated	0.030	0.625	NA (mean 0.645)	1.000	0.800	0.246	1.000	NA	0.002		8
Manually filed	1.000	0.561	0.700	1.000	0.884	0.443	0.923	0.142	0.142	2	2
90°C 1 min	0.563	0.788	0.571	1.000	0.860	0.456	0.899	0.089	0.089	5	5
95°C 1 min	0.714	0.733	0.500	0.995	0.908	0.456	0.921	0.101	0.101	3	3
100°C 1 min	0.863	0.682	0.625	0.989	0.878	0.484	0.916	0.144	0.144	1	1
Boil 0.5 min	1.000	0.323	0.836	0.979	0.871	0.539	0.779	0.097	0.097	4	4
Boil 1 min	1.000	0.260	0.619	0.957	0.914	0.566	0.841	0.067	0.067	6	6
Boil 2 min	1.000	0.189	0.667	0.726	0.961	0.581	0.880	0.045	0.045	7	7
**Low elevation trees**											
Not treated	0.022	1.000	NA (mean 0.597)	1.000	0.500	0.250	1.000	NA	0.002		8
Manually filed	1.000	0.409	0.748	1.000	0.917	0.478	0.939	0.126	0.126	2	2
90°C 1 min	0.333	0.747	0.500	1.000	0.824	0.518	0.965	0.051	0.051	4	4
95°C 1 min	0.456	0.714	0.500	1.000	0.892	0.489	0.967	0.068	0.068	3	3
100°C 1 min	0.663	0.719	0.625	1.000	0.938	0.505	0.950	0.134	0.134	1	1
Boil 0.5 min	1.000	0.202	0.723	1.000	0.833	0.460	0.767	0.043	0.048	5	5
Boil 1 min	1.000	0.157	0.500	1.000	0.929	0.521	1.000	0.038	0.038	6	6
Boil 2 min	1.000	0.078	0.583	1.000	0.857	0.719	0.833	0.023	0.023	7	7
**Mid elevation trees**											
Not treated	0.044	0.500	NA (mean 0.673)	1.000	1.000	0.125	1.000	NA	0.002		8
Manually filed	1.000	0.547	0.700	1.000	0.745	0.407	0.857	0.099	0.099	5	5
90°C 1 min	0.533	0.911	0.500	1.000	0.817	0.502	0.845	0.084	0.084	6	6
95°C 1 min	0.769	0.791	0.500	0.986	0.944	0.481	0.913	0.124	0.124	3	3
100°C 1 min	0.922	0.656	0.714	0.966	0.797	0.484	0.880	0.142	0.142	2	2
Boil 0.5 min	1.000	0.458	0.867	0.964	0.782	0.632	0.952	0.180	0.180	1	1
Boil 1 min	1.000	0.378	0.571	0.912	0.853	0.676	0.893	0.101	0.101	4	4
Boil 2 min	1.000	0.322	0.857	0.517	0.966	0.650	0.857	0.077	0.077	7	7
**High elevation trees**											
Not treated	0.022	0.500	NA (mean 0.693)	1.000	1.000	0.368	1.000	NA	0.003		8
Manually filed	1.000	0.727	0.725	1.000	0.969	0.493	0.951	0.239	0.239	1	1
90°C 1 min	0.830	0.705	0.700	1.000	0.952	0.386	0.900	0.135	0.135	4	4
95°C 1 min	0.923	0.692	0.714	1.000	0.921	0.444	0.881	0.165	0.165	3	3
100°C 1 min	1.000	0.670	0.550	1.000	0.951	0.529	0.911	0.169	0.169	2	2
Boil 0.5 min	1.000	0.308	0.826	0.976	0.964	0.532	0.640	0.082	0.082	5	5
Boil 1 min	1.000	0.244	0.708	1.000	1.000	0.498	0.682	0.059	0.059	6	6
Boil 2 min	1.000	0.167	0.625	1.000	1.000	0.463	0.938	0.045	0.045	7	7

*SI is a product of values for imbibition percentage, germination percentage, germination time, abnormalities, seedling growth, and seedling survivorship to determine the optimal scarification treatment while simultaneously accounting for all processes. SI_m_ used mean for germination time across treatments for untreated seeds as germination time could not be calculated. Values for each constituent process and SI range from 0 to 1 and treatment with the highest value indicates the optimal treatment*.

Among different processes, scarification treatment had the largest impacts on imbibition and germination, with the largest CV of 0.44 for both across treatments (Table 2). In contrast, scarification treatment had relatively small impacts on early mortality and seedling survivorship, with a CV of 0.053 and 0.073, respectively. CV for germination time (0.16), abnormalities (0.10), and seedling growth (0.22) were in between these processes.

## Discussion

### Intraspecific Variation in the Effectiveness of Scarification Treatments

Effectiveness of scarification treatments in causing imbibition differed by elevation of mother trees in koa seeds, reflecting large intraspecific variations in various morphological and physiological traits (Whitesell, 1984; Daehler et al., 1999; Ares et al., 2000; Ishihara et al., 2017). Supporting our hypothesis, seeds from lower elevations required more intense scarification treatments than seeds from higher elevations. Most studies in non-crop species have focused only on the overall effectiveness of scarification conditions and do not account for intraspecific variations (but see Van Haverbeke and Comer, 1985 for red cedar; Boyle and Hladun, 2005 for another species in Fabaceae; Burrows et al., 2009 and Aref et al., 2011 for other species in *Acacia*). In koa, differences in sensitivity to hot water exposure are likely due to differences in strength (Ma et al., 2004) or thickness (White, 1908; Burrows et al., 2018) of the water impermeable cuticle layer outside the palisade layer in the seed coat, although few studies exist on intraspecific variation in seed coat characteristics.

Within the genus *Acacia*, substantial variation in seed coat thickness exists (Cavanagh, 1980; Burrows et al., 2018). The water entry area on the seed coat for koa is likely the lens (Burrows et al., 2009, 2018; Gama-Arachchige et al., 2013). Because the seed coat is of maternal origin (Radchuk and Borisjuk, 2014), differences in mother tree environment along our steep elevational gradient might have led to differences in seed coat characteristics that were reflected in sensitivity to hot water exposure (Tapke, 1924). Our results were consistent with previous studies reporting greater physical dormancy for seeds from plants grown under higher temperature but inconsistent in that seeds from trees in wetter conditions showed greater physical dormancy (Hudson et al., 2015; Liyanage and Ooi, 2015; Jaganathan, 2016). Such inconsistency can be explained by the fact that high temperature at low elevation was also associated with higher rainfall and relative humidity on our site. The strength of physical dormancy may also increase with decreasing seed size because of greater palisade layer thickness in the lens fissure relative to seed mass, as shown in another species in Fabaceae (Rodrigues-Junior et al., 2018). Similarly, across different species including *Acacia*, cuticle was also more developed in smaller seeds than larger seeds (White, 1908) and smaller seeds had smaller lens, which may form smaller water gap (Burrows et al., 2018). These observations linked to seed size also agree with our finding that high elevation seeds were more sensitive to hot water exposure because koa seed size increased with increasing elevation (Sugiyama et al. in prep).

### Assessing Processes Beyond Germination Using *SI*

Increasing the intensity of scarification treatment increased the proportion of seeds that imbibed but had negative effects on processes beyond imbibition and germination in koa although many studies focus on the effectiveness of different treatments on germination percentage and germination time (but see Tapke, 1924; González-Castañeda et al., 2004). Effects of increasing the intensity in treatment were generally linear for imbibition and abnormalities, but negative effects on germination percentage were not. Contrary to our expectations, manually filing seeds generally did not result in the highest germination percentage across all treatments, similar to the result in congeners when seeds were manually chipped using a knife (Clemens et al., 1977) or filed using sandpaper (de Zwaan, 1978). Koa seeds do not have physiological dormancy and we only sowed seeds that imbibed, so the difference in dormancy level does not explain the difference in germination. As evident from the fact that not all manually filed seeds imbibed after the first attempt, we carefully filed seeds, which was unlikely to have damaged or killed the seeds. Because untreated seeds that imbibed also showed similar germination levels, specifically, exposure to 90–100°C water rather promoted germination although boiling seeds substantially reduced germination as observed in congeners including the closely related species *Acacia melanoxylon* (Clemens et al., 1977; de Zwaan, 1978; Le Roux et al., 2014). Koa is adapted to fire (Scowcroft and Wood, 1976; Baker et al., 2009) and heat, and this may have promoted germination along with seedling growth. Although we sterilized all seeds, it is also possible that hot water might have reduced seed-borne pathogens, as observed in other species (Nega et al., 2003; Bennett and Colyer, 2010; McDonnell et al., 2012).

The type of scarification treatment on koa seeds had the largest impacts on imbibition and germination, the first two processes directly affected by different treatments. It was possible that differences set at early processes may have led to even greater variations in later processes, but these were largely independent of one another and variation among treatments was smaller after germination. This was likely because seeds that were severely impacted by a treatment were all killed without germinating and were not available for the subsequent measurements, as evidenced by relatively low abnormalities and early mortality. Various germination indices considering germination percentage and germination time have been developed and used to compare different scarification treatments (Kader, 2005). If we had used a germination index instead to rank different treatments, the rank would have been different because the rank for germination or germination time and *SI* rarely matched. In contrast, untreated koa seeds germinated at the same level as manually filed seeds but *SI* values for this treatment consistently ranked lowest due to low imbibition and seedling growth.

Processes and responses beyond germination, such as abnormalities, are rarely recorded in studies comparing scarification treatments (but see Tapke, 1924). Germination is often recorded based on short elongation of the radicle, which may not capture abnormalities. If one were to select a scarification treatment to maximize the number of vigorous seedlings while minimizing the effort, decisions based on an index focused on germination would be misleading unless radicle emergence predicts sound seedling development (Matthews et al., 2018). Imbibition is incorporated in germination in many studies but in a species such as koa where imbibing seeds is laborious and a prerequisite for germination, specifically accounting for imbibition using an index like *SI* can help make decisions. If only a small proportion of seeds imbibe with exposure to hot water after the first attempt, one may decide to boil the seeds for 0.5 min once, even at the cost of reducing germination, to reduce the effort for imbibing the seeds.

### Effects of Repeated Exposure to Hot Water

Little is known about the effects of repeated exposure to hot water because most previous research did not repeat the treatment when seeds did not imbibe (but see Baskin et al., 2007). However, in practice, scarification treatments need to be repeated or altered to germinate hard seeds like koa. Repeating the treatment is recommended in koa when seeds do not imbibe after exposure to near-boiling water (Wilkinson and Elevitch, 2003), which in fact did not reduce germination. We assumed little negative post-germination impact of hot water on seeds as long as the seed coat was intact (Tapke, 1924). However, repeated exposure had overall negative effects beyond germination, especially on early mortality and seedling growth, and when seeds were boiled. Abnormalities and early mortality both indicate damage to seeds that managed to germinate but the effects of repeated exposure and the intensity of the treatment were different for them. Abnormalities were relatively rare but when observed, the vast majority occurred after the first attempt and increased with increasing treatment intensity. This was likely because if seeds that imbibed with intense treatment after the first attempt managed to germinate but with abnormalities, seeds that had to be treated multiple times presumably died without germinating. In contrast, early mortality increased with the number of attempts but not by the intensity of the treatment. Repeated heat exposure may have gradually increased lipid peroxidation and decreased activities of scavenger enzymes of free radical and peroxide in seeds (Hsu et al., 2003), which can lead to early mortality of seedlings.

Repeated treatments could have increased the amount and size of microscopic fractures on the seed coat as lipids were lost during exposure to hot water (Ma et al., 2004; Zeng et al., 2005). Those microscopic fractures, whose locations on seeds also determine the intensity of the damage (Tapke, 1924), likely increased significantly when seeds were boiled and resulted in heat or imbibition damage (Schelin et al., 2004; Boyle and Hladun, 2005) when seeds were immediately cooled. The integrity of the seed coat may explain why seeds were more likely to imbibe only partially after the first attempt. Seeds exposed to hot water once may have incurred non-visible fractures (Burrows et al., 2018), which led to higher likelihood of seeds fully imbibing after multiple attempts. If seeds that eventually imbibed fully were exposed to hot water with microscopic fractures longer than seeds that were partially imbibed with fewer fractures, fully imbibed seeds would have resulted in lower germination. Whether seeds were likely to imbibe fully or partially, a characteristic likely associated with the property of the seed coat, was largely determined by the identity of mother tree. Seeds from mid elevation trees tended to have a greater proportion of seeds imbibing only partially. Such tendency may have resulted from a more fracture-resistant seed coat in response to wet environments in mid elevation (Supplementary Figure 4) and help to explain why boiling seeds for 0.5 min resulted in the highest *SI* although higher physical dormancy in wetter environments is rather exceptional (Hudson et al., 2015; Jaganathan, 2016). Whether scarification treatments would have any long-term effects on seedling performance beyond the seven months of the study period, such as eventual reproduction (Tapke, 1924), requires further study. Yet, koa seedlings are usually outplanted within 12-18 weeks (Wilkinson and Elevitch, 2003) so we observed the entire period during which any scarification treatment effects would have appeared on typical koa seedlings in nurseries.

### Recommended Approaches for Scarifying Koa Seeds

Scarifying koa seeds using hot water once was not very effective unless seeds were boiled, but boiling seeds had largely negative effects on later processes. Previously recommended treatment of soaking koa seeds in 90°C water for 1–3 min (Wilkinson and Elevitch, 2003; Elevitch et al., 2006) would have resulted in fewer than 25% of seeds imbibing after the first attempt or fewer than 60% even after 15 attempts and even less if seeds were from low elevations. We used a ratio of 20 parts water to one-part seeds instead of 5–10 parts (Wilkinson and Elevitch, 2003; Elevitch et al., 2006) so this estimate is likely to be even lower with a 5–10 parts ratio. We did not change exposure times to 90 to 100°C water because we did not see any difference in pre-trials, which included exposure to 90, 95, and 100°C water for 2 or 3 min. This is likely because water temperature quickly decreased with time. If one uses a water bath to maintain temperatures between 90 and 100°C, increasing exposure time may prove to be more effective and provide a more promising alternative. Until this is confirmed, we recommend considering using an electric seed scarifier machine for mechanically treating large quantities of koa seeds. If the sample size of seeds is relatively small, one can expose the seeds to 100°C water for 1 min once or twice and manually file the remaining non-imbibed seeds, which will still reduce the manual effort. Non-visible damage can be detected by soaking seeds in room temperature water overnight before treating them with hot water and is always recommended to avoid any unnecessary treatments.

Seeds collected from different parts of the island may show different responses, which could have led to different previously recommended scarification conditions. Even within Hawai'i Island, seeds from different geographic regions will likely show some difference as seen by large intraspecific variations we have observed, which were not simply captured by elevation although many environmental variables are highly correlated with elevation (Giambelluca et al., 2013). Based on the results of imbibition, germination, and germination time from pre-trials, we boiled seeds for 0.5 min for all trees beyond the nine trees that were subjected to all treatments. However, pre-trial seeds were from a different geographic region on Hawai'i Island, which showed fewer negative effects from boiling for 1 min, and greater phyllode development (mean 8.9 ± 14.0 phyllodes although they had up to 38 more growing days). Tolerance to boiling varies substantially within *Acacia* (Larsen, 1964; Clemens et al., 1977; Doran et al., 1983), even more so considering intraspecific variation. It is advised to start with pre-trials (Wilkinson and Elevitch, 2003; Elevitch et al., 2006) but also to ensure that seeds for pre-trials are representative of seeds for the actual trials.

### Potential for Screening Seeds and Seedlings

By recording treatment responses, we observed a potential for screening promising seeds and/or seedlings. For example, although seeds that imbibed without treatment germinated at the same level as manually filed seeds and survived well, those seedlings grew poorly. The same was true for seeds that did not imbibe after 15 attempts and were subjected to manual filing, although they germinated at above average levels. If obtaining vigorous seedlings is the goal, these seeds could be screened and excluded whereas if assessing seed viability by germination is the primary interest or seeds are scarce resources and seeds and genetic diversity are to be maximized, these seeds would be included. High quality seeds may also be screened based on traits such as seed mass (Sugiyama and Peterson, 2013; Calvo et al., 2016) and germination time (Matthews et al., 2018; Luna-Nieves et al., 2019), which can be correlated (Ghassali et al., 2012; Calvo et al., 2016; Luna-Nieves et al., 2019). However, we did not detect any effect of seed mass for koa (data not shown). Overall, we do not recommend boiling seeds or repeating the treatment too many times in koa but if boiling treatment for 0.5 to 2 min is to be repeated, seeds that did not imbibe after four to six attempts of boiling never germinated so those seeds could be discarded without further repeating treatments.

Considering that most seeds used in restoration projects are collected in natural forests (Jalonen et al., 2018), seed waste not only leads to inefficient and expensive practices but also to waste of natural resources, which may impose negative consequences on natural regeneration and wildlife populations (Murali et al., 1996; Peres et al., 2003; Broadhurst et al., 2008, 2016). Although predicting high seed quality and seed sources may not be easy (Luna-Nieves et al., 2019), future work on improving predictability of screening promising seeds and/or seedlings through scarification treatments may help to reduce seed waste and improve operation efficiency.

## Data Availability Statement

The raw data supporting the conclusions of this article will be made available by the authors, without undue reservation.

## Author Contributions

AS contributed to conception and design of the study, organized the data, performed experiments and statistical analyses, and wrote the first draft of the manuscript. All authors contributed to manuscript revision, read, and approved the submitted version.

## Funding

This work was funded by the van Eck Forest Foundation at Purdue University and USDA National Institute of Food and Agriculture, McIntire Stennis project IND011535.

## Conflict of Interest

The authors declare that the research was conducted in the absence of any commercial or financial relationships that could be construed as a potential conflict of interest.

## Publisher's Note

All claims expressed in this article are solely those of the authors and do not necessarily represent those of their affiliated organizations, or those of the publisher, the editors and the reviewers. Any product that may be evaluated in this article, or claim that may be made by its manufacturer, is not guaranteed or endorsed by the publisher.
