# Supplementary material for: Intraspecific Variation Along an Elevational Gradient Alters Seed Scarification Responses in the Polymorphic Tree Species Acacia koa
Source: Front Plant Sci. 2021 Nov 4;12:716678. doi: 10.3389/fpls.2021.716678 (PMC8601391; doi:10.3389/fpls.2021.716678)
Supplement: Supplementary file 1 [file Data_Sheet_1.pdf]

frontiers

Supplementary Material

Intraspecific variation along an elevational gradient alters seed scarification responses in the polymorphic tree species *Acacia koa*

Anna Sugiyama, James B. Friday, Christian P. Giardina, Douglass F. Jacobs

Experiment type	Source	Collection year	Tree no.	Elevation (m)	*N*
Full (low elevation)	Seed bank	2014	AK43	308	218
	Seed bank	2014	AK44	308	280
Full (low elevation)	Seed bank	2014	AK42	309	219
	Seed bank	2014	AK41	316	39
	Collected	2018	AK27	333	40
	Collected	2018	AK62	333	280
Full (low elevation)	Collected	2018	AK26	335	461
	Seed bank	2014	AK60	481	40
Full (mid elevation)	Collected	2018	AK38	701	220
	Collected	2018	AK10	810	40
	Collected	2018	AK9	812	41
Full (mid elevation)	Collected	2018	AK8	899	220
	Collected	2018	AK7	902	40
	Collected	2018	AK31	908	40
	Collected	2018	AK30	920	40
	Collected	2018	AK3	931	40
	Collected	2018	AK4	933	40
Full (mid elevation)	Collected	2018	AK5	937	221
	Collected	2018	AK6	937	39
	Collected	2018	AK2	988	41
	Seed bank	2014	AK51	1204	40
	Seed bank	2014	AK52	1204	40
	Seed bank	2014	AK54	1204	40
	Seed bank	2014	AK50	1206	40
	Seed bank	2014	AK53	1210	40
Pre-trial	Purchased from a nursery	2018	AK61	1372	230
	Seed bank	2016	AK55	1451	40
	Seed bank	2014	AK56	1451	40
	Seed bank	2014	AK59	2005	280
	Seed bank	2014	AK58	2005	40
Full (high elevation)	Seed bank	2014	AK57	2008	220
	Seed bank	2014	AK49	2019	40
	Seed bank	2014	AK48	2020	280
	Seed bank	2014	AK47	2031	40
	Seed bank/Collected	2015/2018	AK19	2035	40 each
	Collected	2018	AK20	2038	40
Full (high elevation)	Seed bank	2014	AK18	2040	460
	Seed bank	2014	AK46	2079	40
Full (high elevation)	Seed bank	2014	AK45	2103	220

Table 1 List of mother trees ordered by their elevations and sample sizes of seeds used in this study.

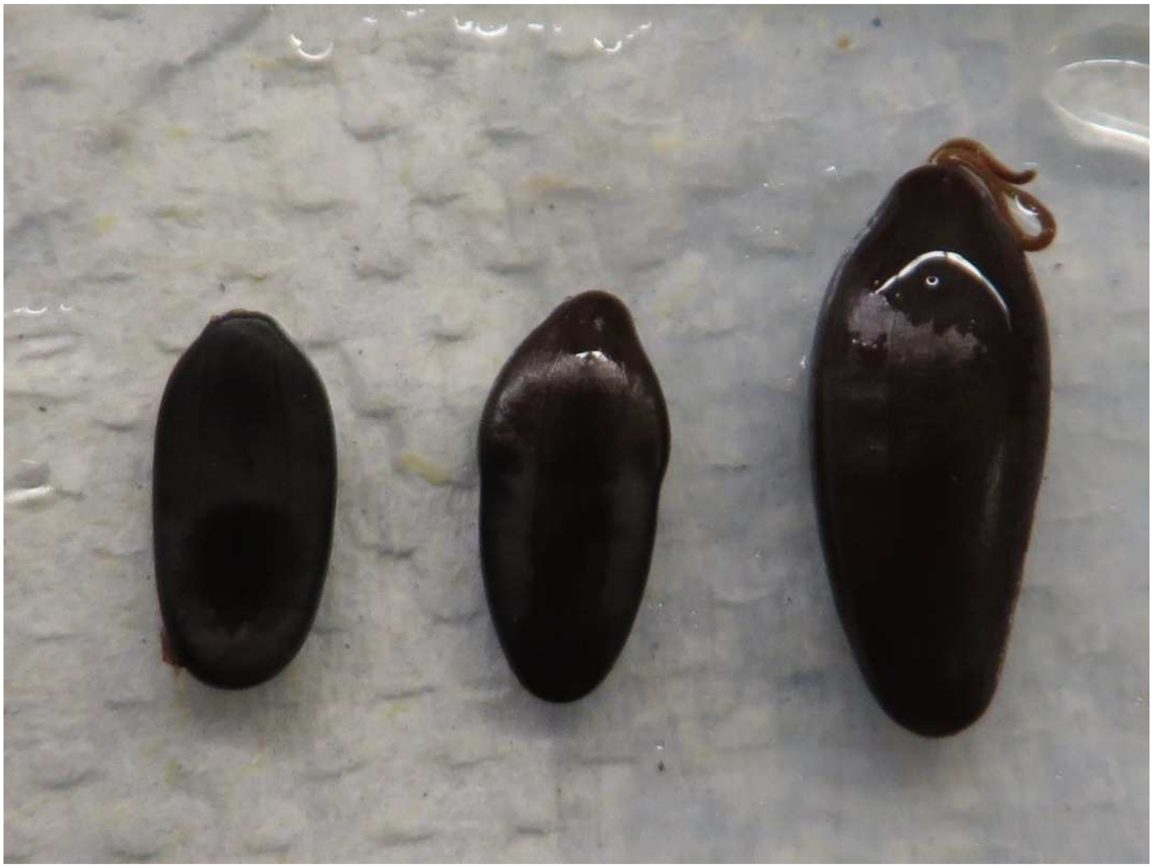

Figure 1 Koa seeds that are intact (left), partially imbibed (middle), and fully imbibed (right).

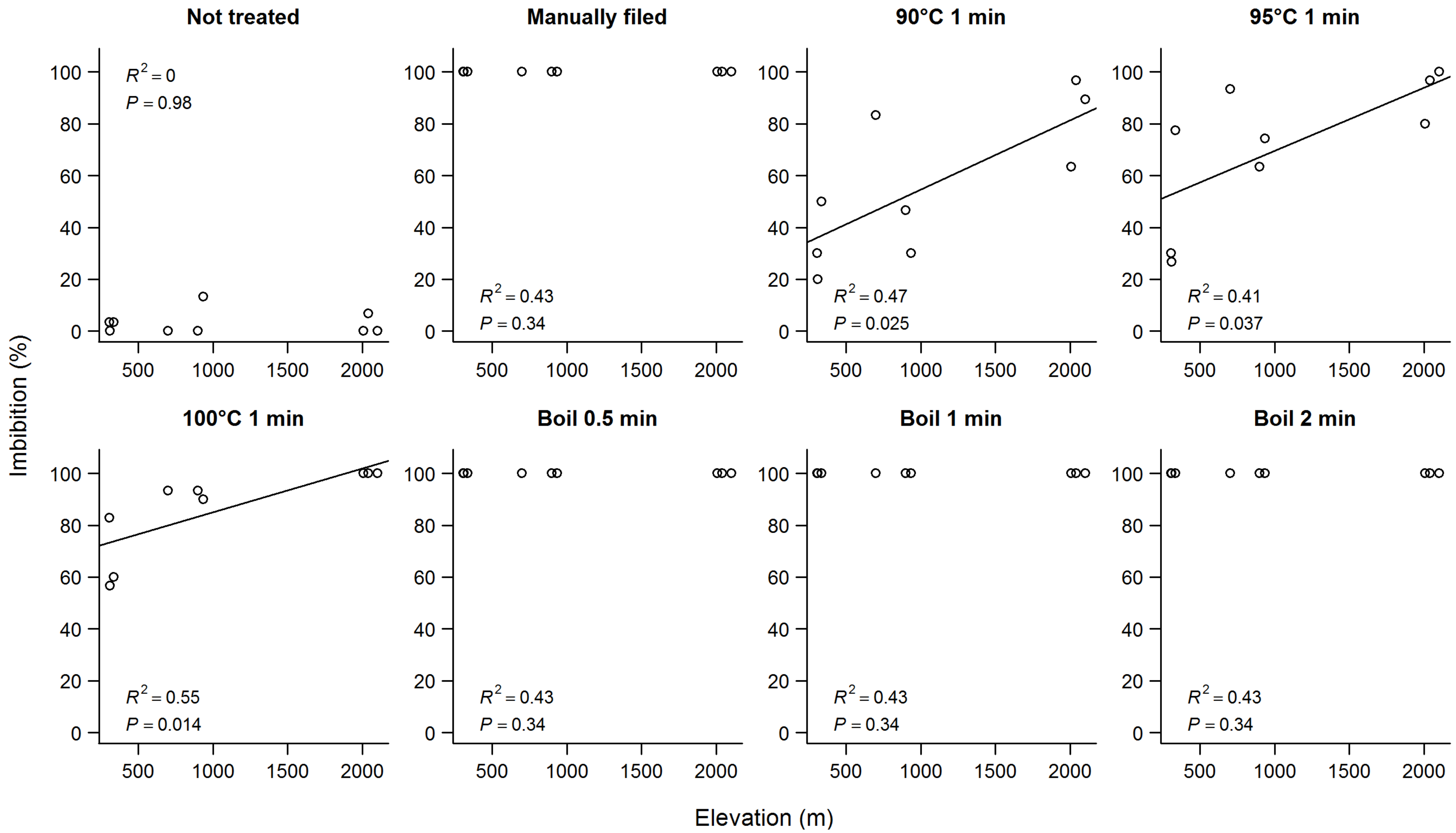

Figure 2 Imbibition percentage of koa seeds and elevation of mother trees after up to 15 applications of a treatment combined. When seeds did not imbibe after the 15th attempt, we manually filed them, which resulted in 100% imbibition (these results are excluded here). Data for each subpanel are from nine trees subjected to all scarification treatments.

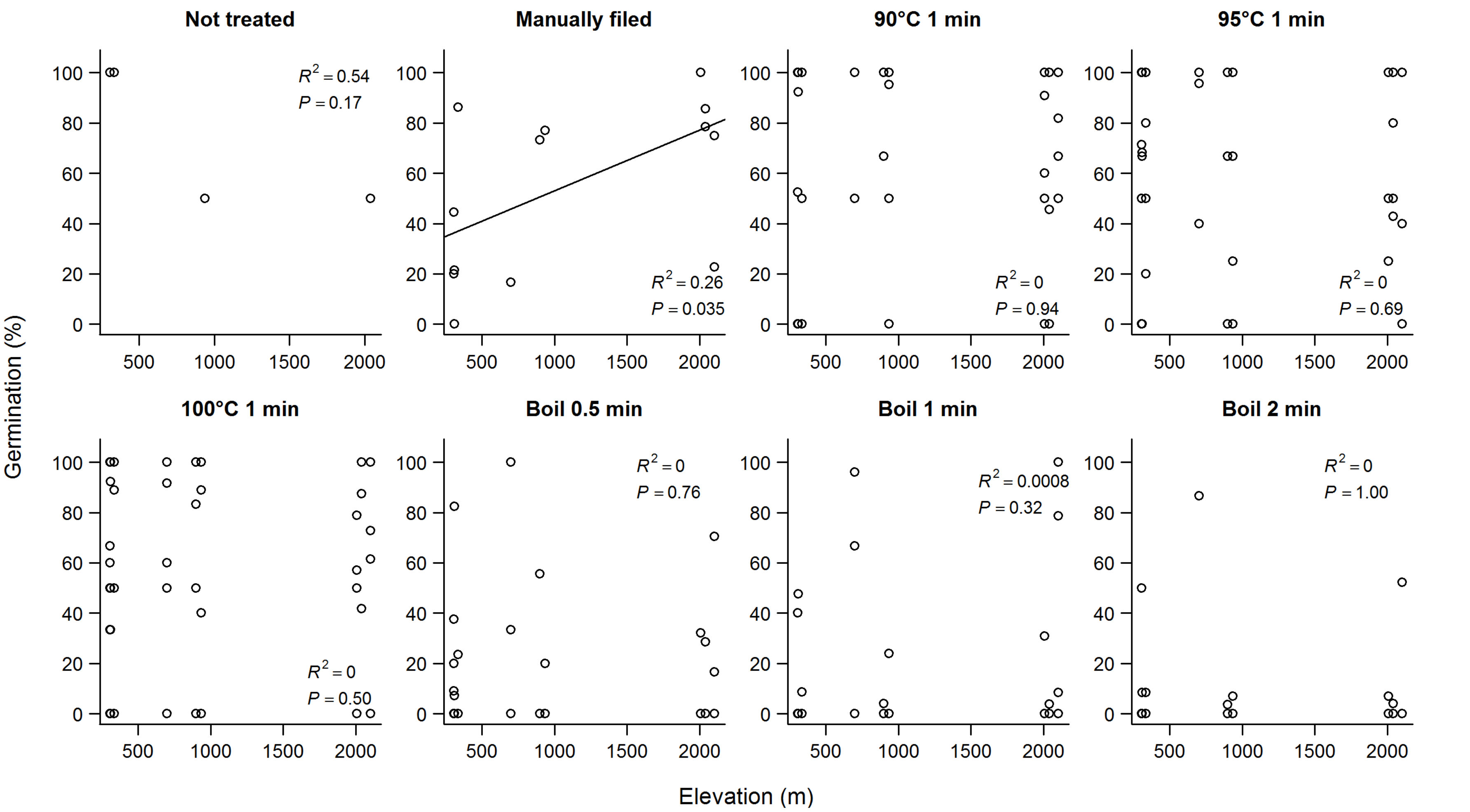

Figure 3 Germination percentage of koa seeds and elevation of mother trees after up to 15 applications of a treatment combined. When seeds did not imbibe after the 15th attempt, we manually filed them (these results are excluded here). Data for each subpanel are for seeds from nine trees subjected to all scarification treatments.

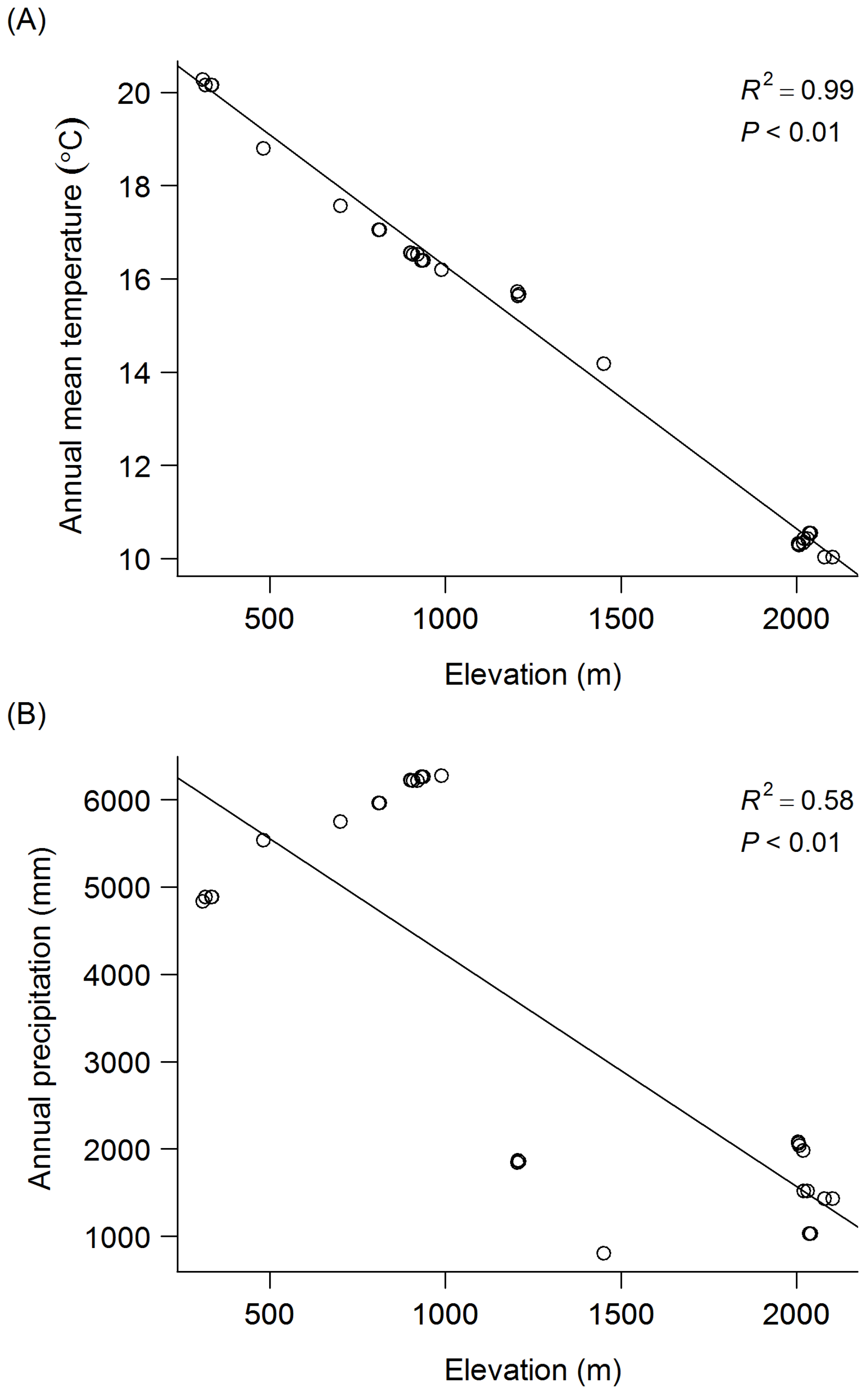

Figure 4 Relationship between elevation and (A) annual mean temperature and (B) annual precipitation for koa trees used for full experiments. We obtained environmental variables for each mother tree from Giambelluca *et al.* (2013).

Supplementary Material References

Giambelluca, T.W., Chen, Q., Frazier, A.G., Price, J.P., Chen, Y.-L., Chu, P.-S., et al. (2013). Online Rainfall Atlas of Hawai'i. *Bulletin of the American Meteorological Society* 94, 313-316. doi: 10.1175/BAMS-D-11-00228.1.
